# Comment on Wang et al. Simufilam Reverses Aberrant Receptor Interactions of Filamin A in Alzheimer’s Disease. *Int. J. Mol. Sci.* 2023, *24*, 13927

**DOI:** 10.3390/ijms26062480

**Published:** 2025-03-11

**Authors:** Keith Fluegge, Kyle Fluegge

**Affiliations:** Institute of Health and Environmental Research, Columbus, OH 43216, USA; kyle.fluegge@gmail.com

The authors have published their findings that simufilam, a small novel molecule suggested for clinical use in Alzheimer’s disease (AD), restores a deleterious conformation of filamin A (FLNA), which is a large intracellular scaffolding protein, as indicated by changes in isoelectric focusing points [1]. The isoelectric point (pI) is the pH at which a molecule carries no electrical charge. Changes in isoelectric focusing points can also indicate more than just phosphorylation or mechanical stress. Proteolytic cleavage and subsequent changes in protein length can also affect the balance of charges [2], and the authors briefly mention cleavage as a possible interaction in characterizing mutant FLNA. The co-immunoprecipitation experiments use FLNA antibody that is specific to various epitopes on the C or N-terminus. There is the possibility that these epitopes exist on both wild-type FLNA and fragmented protein segments. These experiments prove that FLNA has been altered, but whether this entails a conformational change is not certain.

This consideration is minimized, though, when the authors postulated in their discussion that amyloid beta1–42 (Aβ42) drives this potential altered conformation. If this hypothesis were correct, patients with moderate and/or severe dementia should see an observed clinical benefit from taking simufilam, owing to a greater amyloid burden as the disease progresses. Indeed, patient screening for clinical trials measuring simufilam’s effectiveness in AD involves assessing amyloid pathology. However, on Cassava Sciences’ website, the unpublished phase 2 clinical data thus far suggests that only mild AD cases experience a therapeutic benefit of simufilam [3], indicating that the hypothesis that Aβ42 drives a conformational change in FLNA, which simufilam reverses, is not correct. Rather, the clinical data thus far would suggest that mutant FLNA appears before any amyloid deposition and aggregation, and not after. This is a critical observation because the recent ReThink-ALZ phase 3 readout indicated that simufilam did not improve or even halt cognitive loss in AD. If there is a clinical benefit to simufilam, it may not be readily observable in already diagnosed AD populations with evident amyloid pathology.

**Figure 1:**
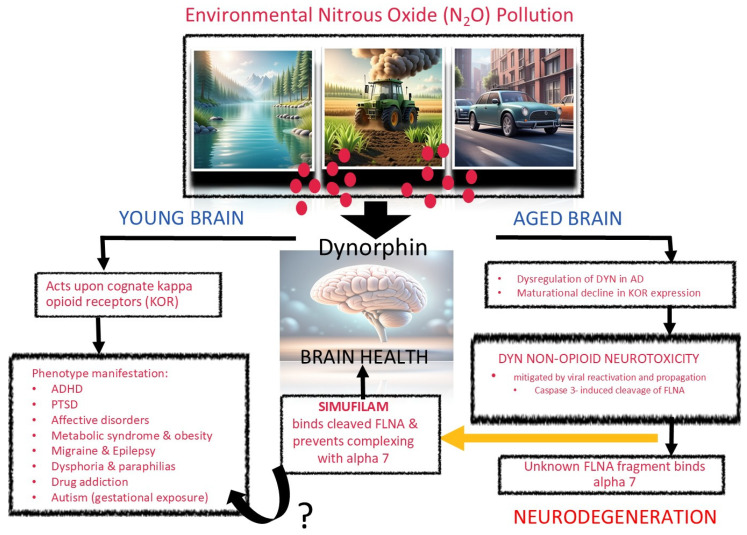
The figure proposes a neurodegeneration cascade induced by environmental nitrous oxide (N_2_O) pollution. N_2_O is an air pollutant that predominately emanates from synthetic nitrogen use in agriculture, as well as mobile and stationary combustion sources and bodies of water. Trace levels of the pollutant induce dynorphin (DYN) opioid peptide release in both human and animal brains. In a young brain, these DYN peptides act upon their cognate opioid receptors, principally the kappa opioid receptor (KOR). This agonism is responsible for many negative, maladaptive states that we and many others have previously discussed, including, but not limited to, ADHD [5,6,7,8], PTSD [9], affective disorders [10,11], metabolic syndrome [12], migraine [13], epileptiform activity [14], paraphilias [15,16], and drug addiction [17]. However, in an aged brain, whereupon constitutive KOR expression is markedly reduced, DYN induces a non-opioid neurotoxicity that is mediated by caspase 3 activation and ultimate fragmentation of FLNA. A suspected, as of yet unidentified, FLNA fragment then binds the alpha 7 that uncouples neuronal nitric oxide synthase, creates a redox imbalance, and ultimately triggers BACE1 activation and amyloid deposition. Simufilam binds this uncharacterized FLNA fragment, maintaining alpha 7 integrity and staving off neurodegeneration. Simufilam’s therapeutic window is, therefore, largely bypassed once amyloid pathology is measurable, and cognitive deficit is apparent. Consistent with simufilam’s potential therapeutic effect against neurodegeneration, we also have argued that concomitant viral reactivation and propagation during neurodegeneration (often noted, perhaps wrongly, as a causal factor) can be an endogenous host mechanism to curb DYN toxicity [18]. It is important to note that these pathways (DYN-mediated KOR stimulation and alpha 7 inhibition) are not mutually exclusive. They can co-occur, but the extent to which they do is largely determined by KORs’ maturational decline in constitutive expression. We have published our findings suggesting that ADHD and AD exist on a continuum of brain degeneration over the lifespan [6,7], with main drivers of environmental N_2_O pollution now being independently linked to both conditions [8,19]. Moreover, many of the other KOR-mediated conditions listed are now recognized as premorbid risk factors for neurodegeneration. It is unknown at this time whether simufilam’s restoration of alpha 7 would have any clinical benefit in treating these KOR-mediated phenotypes, although Cassava Sciences has now licensed simufilam to Yale researchers for investigation as a potential treatment for seizures related to rare neurodevelopmental disorders including tuberous sclerosis complex [20]. We would also be interested in assessing simufilam’s therapeutic effect on ADHD symptomatology and whether it is domain-specific. However, in the case of autism spectrum disorders, we have speculated that a heightened DYN/KOR tone developed during gestational exposure to N_2_O pollution [21], characterized by KOR-mediated behaviors like socio-communicative deficits and behavioral stereotypy, provides a level of protection against lifelong N_2_O exposure and subsequent neurodegeneration risk [22].

We believe that our hypothesis aligns more closely with the ex vivo, in vitro, and clinical data on simufilam. That is, simufilam preserves brain health through binding a highly expressed caspase 3-dependent fragment of FLNA (“from 280 kDa to 170, 150, and 120 kDa major N-terminal and 135, 120, and 110 kDa major C-terminal fragments”) [4], one of which binds the α7 nicotininc acetylcholine receptor, uncouples nitric oxide (NO) metabolism, and generates oxidative stress which promotes Aβ42 generation through BACE1 activation. This critical binding, the beginning of an apoptotic cascade, permits aberrant Aβ42/α7 linkages, which may be instrumental to initiating or maintaining other aberrant linkages that the authors have described. Therefore, simufilam’s mechanism of action prevents Aβ42 through binding of this pathologic FLNA fragment and preventing its complexing with α7 and thereby disabling the formation of other aberrant Aβ42-induced linkages, keeping NO metabolism intact and neurodegeneration at bay [5,6] (See Figure 1).

This hypothesis challenges the current view of the authors that simufilam targets an Aβ42-induced altered FLNA conformation. We suggest here that the authors consider this alternative mechanism and seek clinical efficacy in premorbid conditions to AD to capture this early apoptotic signaling event that simufilam likely precludes (i.e., restoration of the α7). Those conditions could include, and perhaps principally, adult ADHD, a neurodevelopmental disorder often diagnosed in childhood that is characterized in part, neurologically, by tau accumulation in addition to the common and well-known behavioral traits of inattention and hyperactivity [5,6,7]. We have previously identified ADHD and AD as a continuum of brain degeneration occurring over the lifespan [5,6,7]. Simufilam could halt a lifespan of neurodegeneration at its initial stage (See Figure 1), and we hope the authors consider this mechanistic insight when reevaluating their phase 3 trials and any other future clinical pursuits for simufilam and its related diagnostic.
